# Targeting phase separation: a new strategy to disrupt the stromal-immune axis in colorectal cancer

**DOI:** 10.1186/s12964-026-02849-5

**Published:** 2026-04-13

**Authors:** Chenhao Fu, Xiang Chen, Hao Yi, Haoyu Huang, Lipeng Zhang, Haonan Huang, Fan Jiang, Fayang Lei, Honglong Yu, Jibiao Liu, Houping Zhang, Kan Dai, Zhixiong Wu, Zhen Zong, Huizi Li, Shengxun Mao

**Affiliations:** 1 Department of Gastrointestinal Surgery, The Second Affiliated Hospital, Jiangxi Medical College, Nanchang University, Nanchang, Jiangxi China; 2 The First Clinical Medical College, Jiangxi Medical College, Nanchang University, Nanchang, Jiangxi China; 3 Department of Breast Surgery, Pingxiang People’s Hospital, Pingxiang, Jiangxi China

**Keywords:** Biomolecular aggregates, Liquid–liquid phase separation, Colorectal cancer, Tumor microenvironment, Stromal-immune axis, Cancer-associated fibroblasts, Immunotherapy, Targeted therapy

## Abstract

Colorectal cancer (CRC), as one of the malignant tumors with a high incidence globally, has a complex tumor micro-environment (TME) that plays a key role in tumorigenesis, progression, and treatment resistance. In recent years, liquid–liquid phase separation (LLPS) of biomolecules has been gradually discovered to participate in regulating the dynamic balance of the stromal-immune axis in CRC as an important mechanism for integrating intracellular and extracellular signals. LLPS drives the formation of pathogenic aggregates, reshaping the functions of cancer-associated fibroblasts (CAFs), endothelial cells (ECs), and immune cells, promoting tumor cells to evade immune surveillance and forming an immunosuppressive microenvironment. Current research reveals the core role of LLPS in the malignant interactions between CRC stroma and immune cells, but how to precisely target these phase-separated aggregates remains a challenge. This article reviews the regulatory mechanisms of the stromal-immune axis mediated by LLPS, focusing on innovative therapeutic strategies based on small molecule inhibitors, protein degradation technologies, and nano-drug delivery systems, aiming to provide new theoretical basis and clinical translation directions for precise immunotherapy of CRC, and to promote overcoming the challenges of treatment resistance and recurrence.

## Introduction

Colorectal cancer (CRC) remains a leading global health burden, with over 1.9 million new cases and nearly 930,000 deaths annually worldwide [1–3], where metastatic disease represents a primary cause of mortality due to limited therapeutic options and inherent resistance [4]. This clinical impasse underscores the critical need to deconstruct the complex tumor microenvironment (TME), where dynamic interactions between cancer cells, stromal components, and immune cells collectively drive progression and immune evasion [5–7].

Central to this malignant synergy is the bidirectional crosstalk between the tumor stroma and the immune compartment, termed the "stroma-immune axis [8–10]. "Cancer-associated fibroblasts (CAFs), a dominant stromal element, remodel the extracellular matrix and secrete a plethora of factors that directly suppress anti-tumor immunity [11–13]. In parallel, immunosuppressive cells—including regulatory T cells (Tregs) and myeloid-derived suppressor cells (MDSCs)—are recruited and activated, further crippling effector immune responses [14–16]. This co-evolved circuit establishes a self-reinforcing, immunosuppressive niche that is a formidable barrier to therapy.

A paradigm shift in understanding such spatial and temporal coordination within cells is provided by liquid–liquid phase separation (LLPS).LLPS is a physicochemical process whereby biomolecules with multivalent interaction domains condense into dynamic, membrane-less organelles or "condensates" [17–19]. These condensates are not static entities; they exist on a material continuum from liquid-like droplets to more solid aggregates, and their functional output is exquisitely context-dependent [20–22]. For instance, the tumor suppressor p53 requires physiological LLPS for its transcriptional activity, whereas mutant p53 can form pathological aggregates. Similarly, TGF-β utilizes LLPS for signal transduction, which can yield tumor-suppressive or pro-fibrotic/immunosuppressive outcomes depending on the cellular context. This dynamic and versatile nature makes LLPS a potent regulatory mechanism in cancer biology [23, 24]. CRC serves as a compelling model to investigate LLPS in oncology due to its well-characterized TME, the established role of the stroma-immune axis, and the growing identification of specific, pathogenic LLPS events [25, 26]—such as PTK6-mediated phosphorylation of HNRNPH1—that drive autophagy and tumor progression [27].

**Fig. 1:**
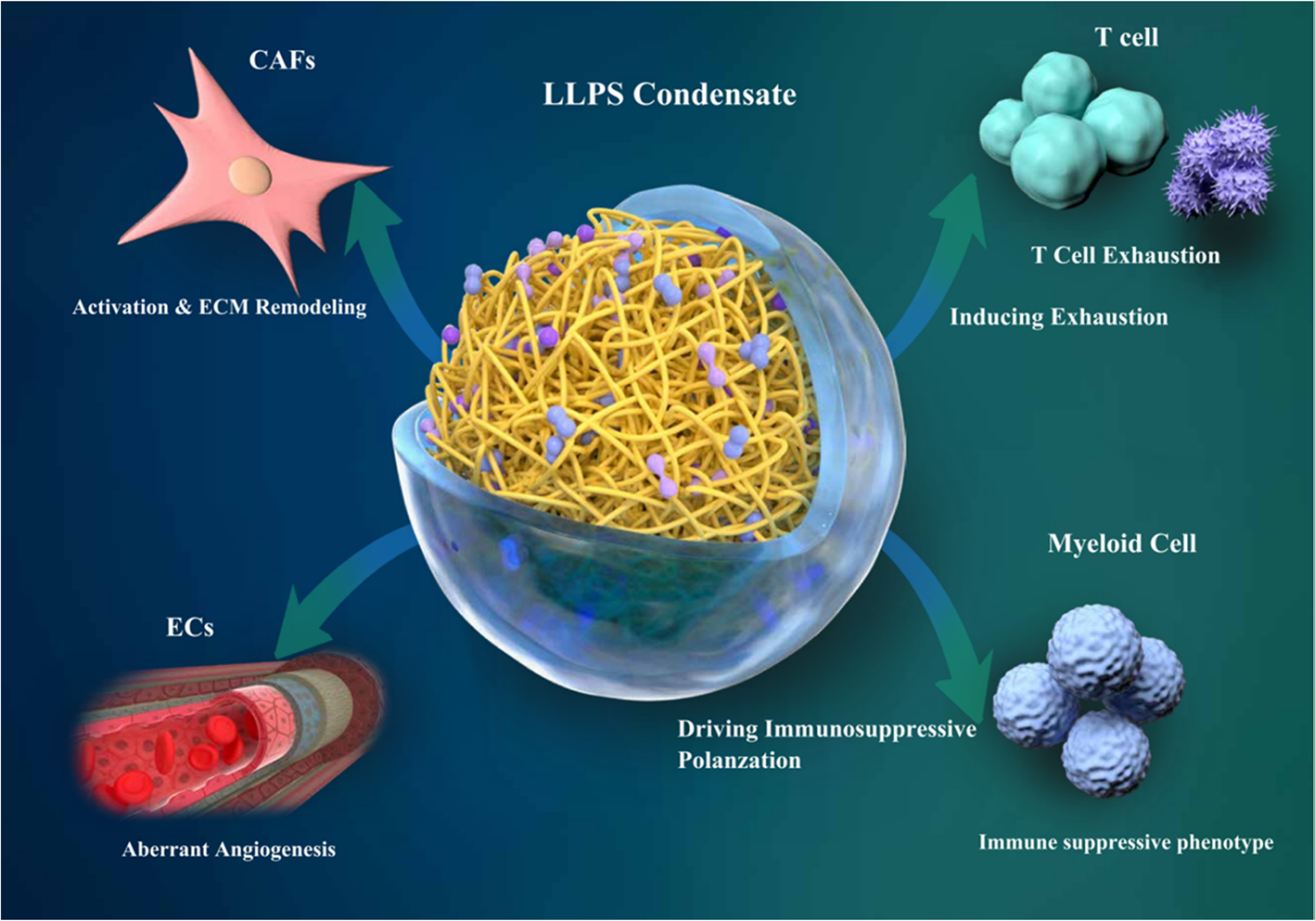
Functional interplay of cellular components in the tumor microenvironment via liquid–liquid phase separation. Liquid–liquid phase separation serves as a core molecular mechanism that drives the malignant remodeling of the colorectal cancer tumor microenvironment through the formation of specific biomolecular condensates in different cell types. CAFs​utilize LLPS to integrate mechanical and biochemical signals, driving the expression of pro-fibrotic and immunosuppressive factors and leading to stromal stiffening. Vascular endothelial cells (ECs)​ employ LLPS to respond to physical cues like matrix stiffness, assembling signal complexes that promote abnormal blood vessel formation. Myeloid cells (e.g., TAMs, MDSCs)​ undergo immunosuppressive polarization directly regulated by LLPS, where metabolites modify the phase separation behavior of key transcription factors (e.g., HIF-1α) to enact immunosuppressive gene programs. T cells​ experience functional changes tightly linked to LLPS, as normal T cell receptor signaling cluster formation relies on phase separation, which is disrupted in the tumor microenvironment, leading to T cell dysfunction. These LLPS-mediated, cell-specific reprogramming events are interconnected and mutually reinforcing, collectively establishing and sustaining a self-perpetuating cycle that supports tumor progression

We posit that LLPS functions as a master regulatory hub that physically encodes and sustains the malignant dialogue of the stroma-immune axis in CRC [28–30]. By forming specific condensates in different cell types, LLPS can integrate diverse inputs (e.g., metabolic signals, mechanical stress, oncogenic mutations) to coordinately reprogram CAFs, dysregulate angiogenesis, and exhaust immune cells [31, 32]. Therefore, targeting these pathogenic LLPS condensates presents a novel therapeutic frontier. This review synthesizes the evidence implicating LLPS as a central driver of stromal-immune dysfunction in CRC and evaluates the promising therapeutic potential of targeting these pathogenic condensates, advancing CRC therapy toward multiaxis strategies that target intercellular communication and microenvironmental regulation (Fig. 1).

### LLPS drives malignant reprogramming in CRC tumor and stromal cells

#### LLPS regulates the activation and pro-tumor functions of cancer-associated fibroblasts (CAFs)

CAFs originate from tissue-resident fibroblasts and acquire a pro-tumor phenotype (such as expressing ECM remodeling components) after activation by TGF-β and PDGF signaling, supporting tumor progression [33–35]. Among them, TGF-β induces the expression of pro-tumor markers through SMAD activation, while PDGF synergistically promotes ECM synthesis and fibrosis [36].

CAFs not only alter the physical environment of tumors by synthesizing and remodeling ECM, but also secrete various proteins and non-coding RNAs containing intrinsically disordered regions (IDRs) that can form extracellular signaling condensates. These paracrine signaling condensates play a role in signal amplification and transmission in the tumor microenvironment, affecting the behavior of tumor cells and immune cells. For example, the SLIT2 protein secreted by CAFs interacts with the ROBO1 receptor and NEK9 kinase, activating cytoskeletal reorganization and transcriptional regulation, promoting the migration and metastasis of tumor cells [37]. CAFs produce pro-inflammatory factors like IL-6 and CCL2. Specifically, CAF-derived IL-6 impairs dendritic cell maturation and T-cell priming via the JAK/STAT3 pathway, thereby weakening the initiation of adaptive antitumor immunity [38]. Furthermore, IL-6, along with chemokines like CCL2, fosters an immunosuppressive niche by inducing the generation of MDSCs and polarizing macrophages towards a pro-tumor M2 phenotype [39–41]. This inflammatory signaling is often amplified through feedforward loops.Tumor-derived metabolites can stimulate CAFs to secrete more IL-6, which in turn activates other stromal components like macrophages, creating a sustained cycle that enhances immunosuppression and tumor immune evasion [42, 43]. By possessing properties of phase-separated liquids, proteins and RNAs in this case are conducive to the aggregation of signaling molecules and the integration of their signaling for the generation of a very dynamic and versatile signaling platform for intra-cellular communication within the tumor microenvironment.

LLPS, an emerging mechanism for intracellular signal integration, is a crucial process for the reciprocal regulation of multiple signaling pathways in CAFs. By inducing and dynamically regulating the nuclear or cytoplasmic enrichment of signaling molecules such as SMADs, JUN/FOS, and YAP/TAZ, LLPS can dramatically increase the sensitivity of CAFs to extrinsic signals and orchestrate gene expression [36, 44]. YAP/TAZ, downstream effectors of the Hippo signaling cascade, regulate extracellular matrix (ECM) and fibrotic properties of CAFs mediated by mechanical signal transduction and sensing of ECM stiffness and composition, thus promoting malignant reorganization of the tumor stroma and supporting immunosuppression [45]. In addition, extrinsic signaling and stimuli such as BRAF inhibitors and ECM stiffness can lead to nuclear reorganization and β-catenin nuclear enrichment in CAFs mediated by the ROCK-cytoskeleton-nuclear force transmission axis, thus invoking an impulsive feedback loop that constantly augments and sustains the pro-tumorigenic activities of CAFs [46]. Current findings highlighted LLPS-mediated signaling integration that can dramatically not only augment the role of CAFs in supporting tumors, but also explore novel therapeutic applications for the specific modulation of CAF activation.

Although in vitro studies have revealed the central role of LLPS in the activation of CAFs (e.g., through the formation of SMADs condensates), the clinical relevance of these findings necessitates cautious assessment. The majority of evidence derives from cell line or organoid models, while in vivo studies (such as real-time imaging utilizing CRC mouse models) remain limited, rendering it challenging to verify the prevalence of LLPS in human tumors. Furthermore, the high heterogeneity of CAFs may result in context-dependent LLPS responses: certain pro-inflammatory CAF subtypes might exhibit reduced reliance on LLPS, an aspect that has not been thoroughly investigated in current research. Future efforts should integrate clinical samples (e.g., patient tissue microarrays) to validate the association between LLPS markers (such as YAP condensates) and disease progression, and address technical hurdles, including resolution constraints in live-cell LLPS detection.While CAFs are central to stromal remodeling, LLPS also critically regulates endothelial cells (ECs), which drive tumor angiogenesis and immune evasion.

#### LLPS mediates abnormal angiogenesis in ECs

Besides CAFs, dysregulated angiogenesis in ECs is further governed by LLPS to enhance the malignant cycle in the TME. Angiogenesis is a classic pathological condition of the colorectal TME, in which ECs play a starring role [47–49]. Mechanical forces, such as the stiffness of the ECM and mechanical stress, regulate ECs’ behavior via LLPS mediated by proteins like DDR1 and Kindlin-2. DDR1 and Kindlin-2 are sensitive to the mechanical properties of the ECM in an LLPS-mediated manner, thus affecting ECs’ migratory, proliferative, and luminary activities to promote the dysangiogenesis of abnormal vasculature in tumors. The transmission of such mechanical signals enables fine-tuned control of the angiogenic process through the formation of dynamic multi-molecular complexes, ultimately leading to the abnormal structure and function of tumor vasculature [50].

Gross neovascularizationnot only hinders infiltration of the immune effector cells, such as T cells, due to physical barriers, but it also represses the immune microenvironment through the emission of diverse signaling factors. LLPS is closely integrated in this process in relation to influencing the abnormalities in the functionality of the malignant vasculature. In this case, it can be observed that the released factor, VEGF-A, originates from the tumor-associated macrophages(TAMs) and closely correlates to the process of LLPS of the ECs in tumors. LLPS may affect vascular permeability or the efficiency of T cells in reaching tumors and thus lead to the generation of immunosuppressive conditions [51, 52].

From the mechanistic perspective, LLPS can dynamically promote the assembly of angiogenic signaling complexes, including those of the VEGF/VEGFR2 axis, and regulate this process in terms of the strength and time course of signaling, thereby directly affecting EC functions. In fact, phase-separation-induced signaling complexes can locally condense signaling molecules and dramatically increase the spatial and temporal precision of signaling cascade events. In addition, EC proliferation rates accelerate, migrational efficiency improves, and lumina become irregular in angiogenic processes, leading to vascular abnormalities in tumors [53–55]. LLPS may regulate EC metabolism and gene expression, further affecting EC sensitivity to mechanical and chemical signals and making LLPS a central regulatory factor in pathological angiogenic processes in tumors [56, 57].

The role of LLPS in the mechanical sensing of ECs is primarily established on simplified in vitro systems (e.g., matrix stiffness assays); however, these models may fail to fully recapitulate the dynamics of the in vivo TME. For example, studies employing 3D culture or live imaging remain limited, and potential differences in LLPS-associated protein expression between human ECs and murine-derived ECs could compromise translational relevance. From a clinical perspective, although vascular abnormality is a hallmark of CRC, direct evidence linking EC LLPS to patient outcomes—such as through immunohistochemical detection of DDR1 condensates—is still lacking. Having established LLPS's role in stromal reprogramming, we next investigate how it directly shapes the immunosuppressive microenvironment by affecting immune cells, thereby completing the stroma-immune circuit.

#### LLPS-driven oncogenic events within colorectal tumor cells

Within colorectal tumor cells, LLPS functions as a central processor that hyperactivates and integrates core oncogenic signaling pathways, directly driving autonomous proliferation, survival, and malignant reprogramming [58]. This is exemplified by its essential role in the dysregulated Wnt/β-catenin cascade. In normal cells, phase separation likely facilitates the assembly of the β-catenin “destruction complex” [59]. In CRC, prevalent APC mutations disrupt this regulatory condensate, leading to the formation of aberrant, hyperactive transcriptional condensates between stabilized β-catenin and TCF/LEF factors on DNA. These pathogenic condensates constitutively drive the expression of c-MYC and cyclin D1, providing a mechanistic basis for the sustained proliferative signal that is a hallmark of CRC.

The oncogenic role of LLPS extends to other pivotal pathways. The Hippo effector YAP/TAZ forms transcriptional condensates that amplify pro-tumorigenic gene expression, often in response to stromal mechanical cues [60]. Simultaneously, the dual nature of LLPS is starkly evident in the p53 pathway: while wild-type p53 utilizes it for tumor-suppressive functions, mutant p53 commonly forms dysfunctional aggregates that promote chemoresistance. Collectively, this network of LLPS-driven events not only fortifies the tumor cell state but also regulates the secretion of factors that remodel the microenvironment, thereby bridging intrinsic oncogenesis with extrinsic immune modulation.

### LLPS shapes the immunosuppressive microenvironment

#### Myeloid cells: LLPS-mediated innate immune evasion

Myeloid cells (including TAMs and MDSCs) play a key role in CRC immune evasion, and the formation of their immunosuppressive phenotype is regulated by LLPS, which mediates this process by affecting the activity and function of transcription factors [61, 62].

**Fig. 2:**
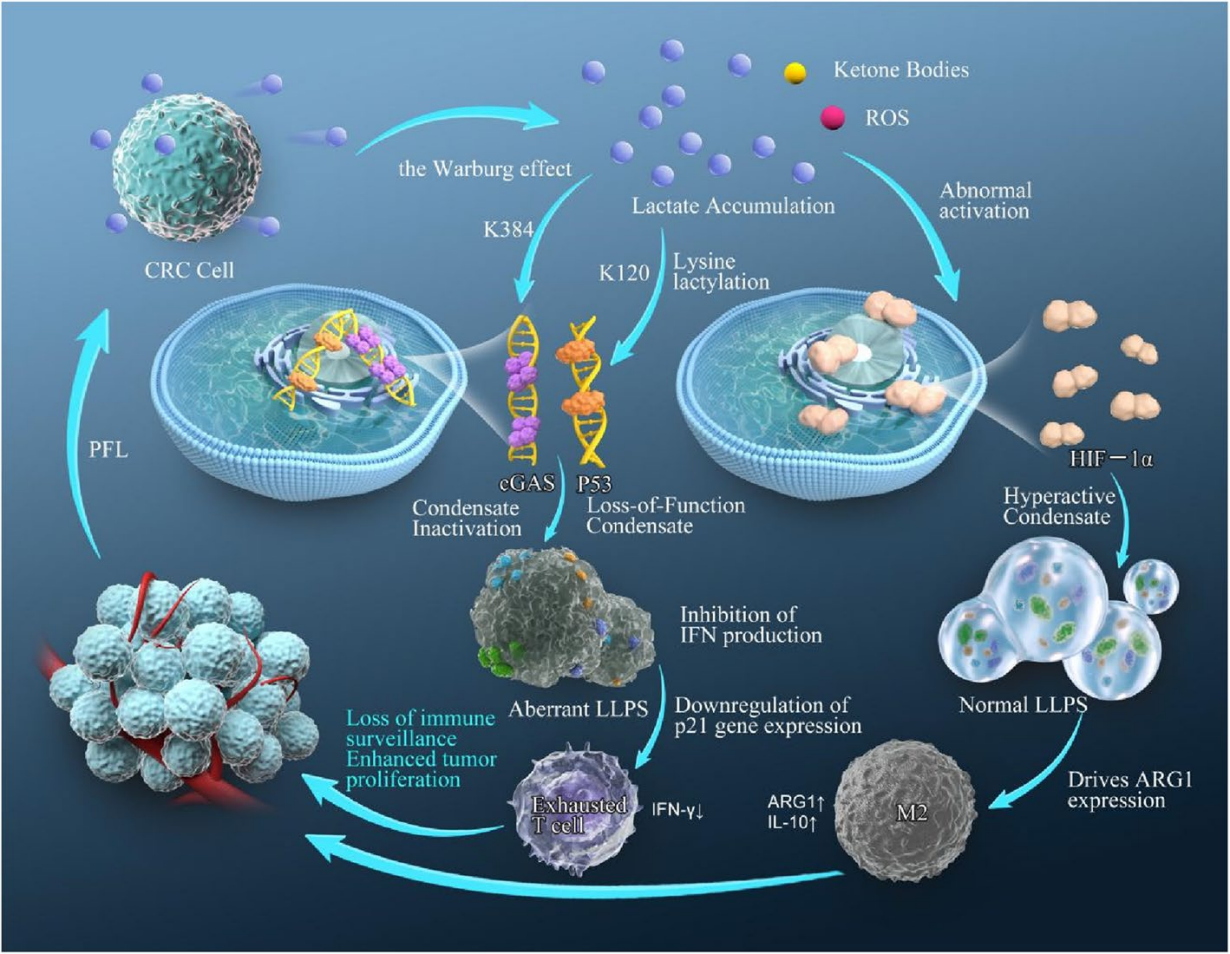
Lactate metabolism reprograms the tumor immune microenvironment by modulating liquid–liquid phase separation. Colorectal cancer cells produce lactate in large quantities via the Warburg effect. Its accumulation in the microenvironment acts as a key metabolic signal that directly regulates the liquid–liquid phase separation of biomolecules through mechanisms such as lysine lactylation, thereby driving immunosuppression. As illustrated, lactate accumulation leads to cGAS condensate inactivation, inhibiting type I interferon production and weakening the innate immune response. Concurrently, it induces the formation of a loss-of-function condensate of p53, downregulating the expression of its target gene p21, and drives the formation of a hyperactive condensate of HIF-1α, promoting the expression of the M2 macrophage marker ARG1. These aberrant LLPS events collectively result in T cell exhaustion (IFN-γ↓) and M2 macrophage polarization (ARG1↑, IL-10↑), ultimately leading to loss of immune surveillance and enhanced tumor proliferation. This model elucidates the lactate-LLPS axis as a central hub connecting tumor metabolism to immune evasion

TAMs typically exhibit an M2-type pro-tumor polarization state in the tumor microenvironment, promoting immunosuppression and tumor progression [63–65]. The accumulation of large amounts of lactate produced by tumor cell glycolysis in the microenvironment can induce the activation of the transcription factor HIF-1α [66] (Fig. 2). Recent studies have further revealed that lactate can promote the LLPS of HIF-1α. This phase separation process facilitates the formation of condensates of HIF-1α in the nucleus, significantly enhancing its transcriptional activity, thereby driving macrophages towards M2 polarization. The phase separation of HIF-1α not only enhances its function as a transcription factor but also promotes the expression of immunosuppressive-related genes, such as IL-10 and ARG1, further inhibiting anti-tumor immune responses. In addition, lactate-mediated HIF-1α phase separation can also affect the metabolic reprogramming of macrophages, promoting their immunosuppressive functionssuch mechanism of phase separation mediated by the transcription factor and metabolic products constitutes a novel approach for understanding the role of TAMs’ pro-tumorigenic polarization and offering possible treatments for TAMs-based immunotherapy [67, 68].

MDSCs exert T-cell function suppression in the presence of molecules such as ARG1 and iNOS [69–71]. Conversely, LLPS controls the expression of such pivotal enzymes: Transcription factors and cofactors increase the transcription of the ARG1 and iNOS genes in phase-separated condensates, leading to increased immunosuppression [72, 73]. LLPS can also play a role in the proliferation of MDSCs, offering novel approaches for therapy.

Metabolic products (such as lactate) regulate the phase separation behavior of proteins through post-translational modifications (PTMs), such as phosphorylation and acetylation, affecting myeloid cell functions [74–76]. Lactate-induced histone acetylation enhances the phase separation of transcription factors, promoting the expression of immunosuppressive genes; glycolytic metabolites regulate the assembly of signaling complexes through phosphorylation, affecting the polarization of TAMs and MDSCs. This metabolic-PTMs-LLPS network supports targeted intervention strategies [67].

#### T cells: functional exhaustion caused by LLPS disruption

T cells, especially CD8 + effector T cells, play a critical role in anti-tumor immunity in the CRC tumor microenvironment. However, various factors in the CRC tumor microenvironment lead to T cell dysfunction or even exhaustion, thereby promoting tumor immune evasion [77]. In recent years, the LLPS mechanism has been found to be involved in the spatial organization of T cell receptor (TCR) signaling and the formation of signaling microclusters, which are crucial for T cell activation [78–81]. Signaling molecules like LAT and GRB2 lead to the formation of signaling microclusters during T cell activation due to liquid–liquid phase separation, thus enhancing TCR signaling and activation responses [82]. Condensates generated in phase-separated systems promote the rapid induction of the immune responses due to the local enrichment of signaling molecules.

In CRC, the assembly of functional signaling condensates is impaired within the tumor microenvironment through multiple mechanisms. These include the presence of tumor metabolites such as lactate and PGE2, the action of the immune checkpoint protein PD-L1, and the direct disruption and exhaustion of T-cell signaling [83]. For example, lactate, a metabolite of the malignant cells, reduces the pH of the T cell environment, thereby affecting the the properties of signaling condensates and hindering T cell activation and proliferation. PGE2 sustains an immunosuppressive environment due to modulation of the expression of cell surface receptors and signaling pathways in effector cells [84, 85]. In addition, increased expression of PD-L1 on the surface of malignant cells directly inhibits T cell activation due to binding of PD-1 on T cells, interfering with signaling condensate integration and inducing T cell exhaustion [86–88].

**Fig. 3:**
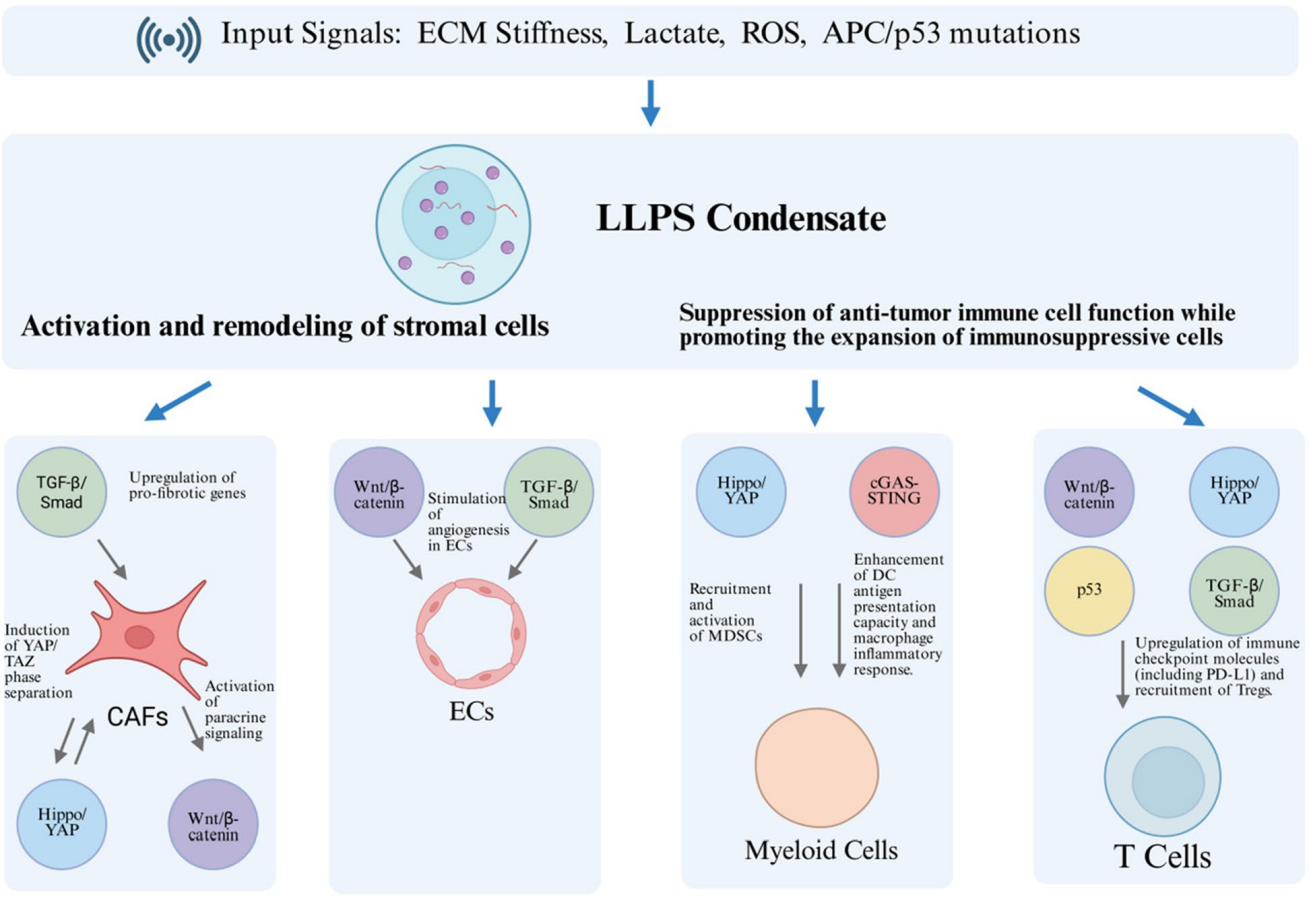
LLPS condensates orchestrate a pro-tumor microenvironment by coordinating stromal activation and immune suppression. Multiple tumor-derived inputs—including ECM stiffness, metabolites (lactate), reactive oxygen species (ROS), and genetic alterations (e.g., APC/p53 mutations)—drive the formation of a dynamic LLPS condensate within the tumor microenvironment. This biomolecular hub centrally coordinates two key malignant processes: Stromal Cell Activation & Remodeling: The condensate fuels Cancer-Associated Fibroblast (CAF)​ activation via Hippo/YAP and Wnt/β-catenin signaling, and upregulates pro-fibrotic gene expression through the TGF-β/Smad pathway. Concurrently, it stimulates Endothelial Cell (EC)-mediated angiogenesis via Wnt/β-catenin and TGF-β/Smad​ activation. Immune Dysfunction & Suppression: The condensate suppresses anti-tumor immunity by recruiting and activating Myeloid-Derived Suppressor Cells (MDSCs)​ and upregulating immune checkpoint molecules​on T cells. It simultaneously promotes the expansion of immunosuppressive populations by enhancing DC antigen presentation and recruiting Regulatory T cells (Tregs)

This metabolic interference extends to innate immune sensors; for instance, lactate-induced lactylation of cGAS disrupts its phase separation and ability to initiate the STING signaling pathway, thereby blunting DNA sensing. This mechanism highlights the role of metabolism-induced PTMs in modulating LLPS, a theme that is also critical in the dysfunction of other signaling pathways discussed next [89] (Fig. 3).

In summary, LLPS orchestrates tumor-immune crosstalk in CRC by establishing a self-reinforcing, immunosuppressive circuit.​ This is achieved through reciprocal interactions between the tumor stroma and the immune compartment. Pathogenic LLPS events within tumor and stromal cells​ actively shape an immune-hostile niche—by creating physical barriers to infiltration and suppressing immunostimulatory signals. This altered microenvironment, in turn, directly subverts functional LLPS within immune cells, such as by disrupting the assembly of critical signaling condensates required for T cell activation. Therefore, LLPS acts as the central mechanistic link that couples stromal reprogramming to immune dysfunction, driving a coordinated program of immune evasion that underpins CRC progression.

### LLPS integrates key oncogenic signaling pathways

#### LLPS regulation of the Hippo/YAP pathway: a bridge between mechanical signals and immune suppression

**Fig. 4:**
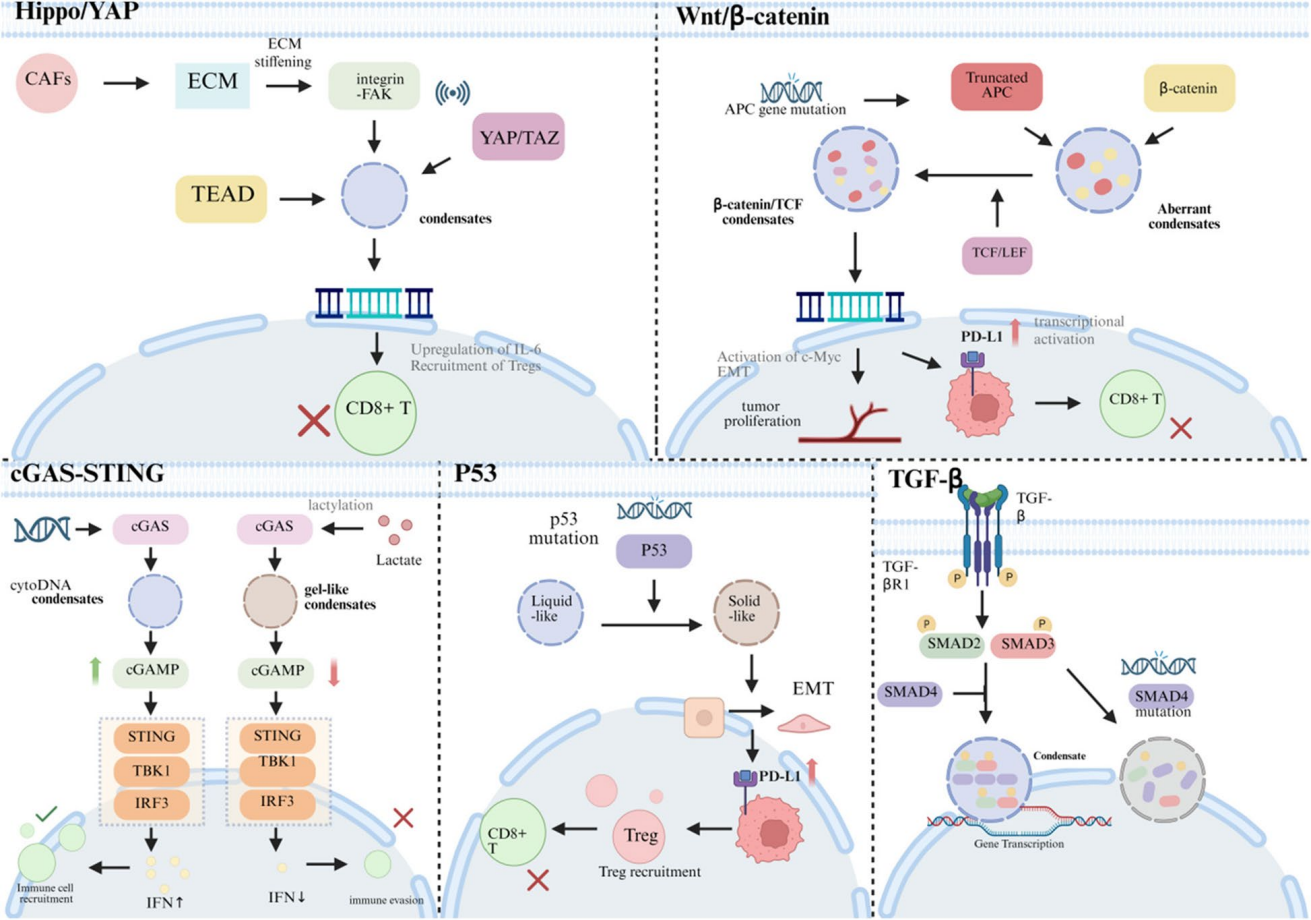
Liquid–liquid phase separation synergistically drives colorectal cancer malignancy by reprogramming five core signaling pathways. Liquid–liquid phase separation serves as a critical master regulatory mechanism that drives colorectal cancer malignancy by concurrently reprogramming five core signaling pathways. Aberrant activation of the Hippo/YAP pathway​ relies on LLPS-mediated formation of YAP/TAZ transcriptional co-activator condensates, promoting pro-proliferative gene expression. In the Wnt/β-catenin pathway, altered LLPS status (transition from "inhibitory condensates" to "active condensates") leads to sustained β-catenin signaling. The immune function of the cGAS-STING pathway​ is strictly dependent on LLPS-formed signaling platforms, which can be suppressed in the tumor microenvironment. The tumor-suppressive function of the p53 pathway​ requires normal phase separation capability, and its disruption results in cell cycle dysregulation. The TGF-β pathway​ dynamically assembles SMAD complexes via LLPS, exhibiting dual roles in tumor progression. These LLPS-interconnected pathways collectively regulate key processes from tumor cell proliferation and immune responses to microenvironment remodeling, establishing a malignant network that supports tumor growth and immune evasion

The Hippo signaling network is identified as the core regulatory circuitry for cell proliferation and differentiation, and its downstream effectors YAP/TAZ are known to be dysregulated in CRC, contributing to oncogenic proliferation and metastases [90–92]. Very recently, it was made evident that LLPS increases the transcriptional expression of downstream genes (for example, CYR61 and CTGF) due to increased nuclear condensate formation of YAP/TAZ and other transcriptional cofactor proteins like SRC-1 and BRD4 [93, 94]. In the TME of CRC, this process is initiated and regulated through biomechanics, for which ECM proteins such as collagen and fibronectin released from CAFs contribute to increased stiffness of the ECM, thereby activating the integrin-FAK signaling cascade and inducing phase separation of YAP/TAZ [95–97]. It has very recently been shown that increased stiffness of ECM triggers the simultaneous condensation of YAP and TEAD transcription factor due to increased multivalency mediated by IDRs, resulting in increased expression of profibrotic and immunosuppressive factors (specifically IL-6 and TGF-β) leading to sustained activation of CAFs, Tregs and MDSCs, inhibiting infiltration and functions of CD8 + T cells in turn [98]. In addition to that, the characteristic dynamic behavior of this LLPS process further enables rapid TME responses of YAP/TAZ condensates in conditions of low oxygen or when met stress levels are increased in the TME, thus further enhancing immunosuppression in turn. Inhibition of this process for therapy emphasizes the need for disruption of YAP condensates, for which several methods have been proposed including use of small molecules (for example, verteporfin and similar molecules or peptide-based molecules that target the IDRs of YAP and thereby counteract the immunosuppressive TME, thus sensitizing CRC tumors toward immune-checkpoint blockade therapy [99, 100] (Fig. 4) (Table 1).

**Table 1:** Dysregulation of key signaling pathways by LLPS in CRC and emerging therapeutic opportunities

Signaling Pathway	Core Role of LLPS	Pathogenic Dysregulation in CRC	Immunomodulatory Consequences in TME	Potential Therapeutic Strategies	Key References
Hippo/YAP-TAZ​	Promotes formation of nuclear YAP/TAZ transcriptional co-condensates with TEAD and co-activators (e.g., BRD4), enhancing transcription of pro-tumorigenic genes	Hyperactivation driven by increased ECM stiffness from CAFs. Constitutive nuclear localization and phase separation of YAP/TAZ sustain a pro-fibrotic and immunosuppressive program	Upregulates secretion of factors (e.g., IL-6, TGF-β) that recruit Tregs/MDSCs and inhibit CD8 + T cell function, fostering an immune-excluded phenotype	Disrupting YAP/TAZ condensates: Verteporfin; Peptide inhibitors targeting YAP-TEAD interaction	[35, 81, 83, 87, 88]
Wnt/β-Catenin​	Orchestrates the assembly of the β-catenin destruction complex (e.g., Axin phase separation). In the nucleus, facilitates β-catenin/TCF transcriptional condensates	APC truncating mutations disrupt the destructive condensate, leading to aberrant stabilization and formation of oncogenic β-catenin/TCF condensates	Drives proliferation and upregulates immune checkpoint molecules like PD-L1 on tumor cells, contributing to T cell exhaustion	Stabilizing Axin (XAV939) or promoting β-catenin degradation: PROTAC molecules	[77, 90, 92, 96]
cGAS-STING​	Essential for cGAS activation upon cytosolic DNA sensing. LLPS drives cGAS-DNA condensate formation, potentiating cGAMP production and downstream IFN signaling	Metabolites (e.g., lactate) induce lactylation of cGAS, impairing its DNA-binding capacity and phase separation, thus blunting innate immune activation	Abrogates the production of type I interferons, reducing dendritic cell maturation and CD8 + T cell priming, enabling immune evasion	STING pathway agonists (e.g., ADU-S100); Inhibitors of lactate production or lactylation	[75, 99, 100, 103]
p53​	Mediates the formation of p53 transcriptional condensates for transactivation of target genes involved in cell cycle arrest and apoptosis	Mutant p53 forms dysfunctional, solid-like aggregates. Lactate-induced lactylation (by AARS1) of wild-type p53 impairs its phase separation and DNA-binding activity	Loss of wild-type p53 function disrupts immune surveillance. Mutant p53 can gain oncogenic functions that promote an immunosuppressive TME	Reactivating wild-type p53: Lactylation inhibitors (e.g., β-alanine), APR-246. Targeting mutant p53 for degradation: PROTACs	[106, 108, 113, 115]
TGF-β/SMAD​	Facilitates the assembly of nuclear SMAD transcriptional complexes through phase separation, regulating genes for differentiation and apoptosis (early) or fibrosis (late)	Exhibits contextual duality. In advanced CRC, sustained signaling promotes SMAD-mediated transcription of pro-fibrotic and immunosuppressive genes in CAFs and T cells	Induces differentiation of Tregs and inhibits cytotoxicity of CD8 + T cells and NK cells, directly suppressing anti-tumor immunity	TGF-β receptor kinase inhibitors (e.g., Galunisertib); Strategies to modulate SMAD phase separation	[116, 119, 122, 123]

#### LLPS regulation of the Wnt/β-catenin pathway: APC mutations and proliferation drive

The Wnt/β-catenin pathway is one of the most frequently mutated signaling pathways in CRC, with approximately 80% of CRC cases harboring APC gene mutations, leading to β-catenin stabilization and nuclear translocation [101]. LLPS regulation of the Wnt/β-catenin pathway is context-dependent. Under normal conditions, LLPS negatively regulates β-catenin by promoting the formation of "degradation condensates"; however, in CRC with APC gene mutations, LLPS aberrantly enhances β-catenin's transcriptional activity [102, 103]. Recent studies have found that APC mutants (such as truncated APC) lose their multivalent interaction capability and cannot effectively assemble degradation condensates, instead forming abnormal condensates with β-catenin, promoting its nuclear accumulation and the expression of downstream oncogenes (such as c-Myc and cyclin D1) [104–106]. In the CRC tumor microenvironment, this leads to increased tumor cell proliferation and influences stromal cells by secreting Wnt ligands (such as Wnt3a). β-catenin condensates recruit transcription factors TCF/LEF in CRC cells, forming super-enhancer-like structures that drive epithelial-mesenchymal transition (EMT) and angiogenesis [107]. At the same time, these condensates regulate immune-related genes, such as the upregulation of PD-L1, thereby inhibiting T cell activity. LLPS regulation is also influenced by oxidative stress: reactive oxygen species (ROS) can modify Axin's IDR, enhancing its phase separation tendency, but APC mutations disrupt this process, contributing to treatment resistance. In response, the latest therapeutic strategies include using Axin stabilizers (such as XAV939) or PROTAC molecules to inhibit β-catenin signaling by restoring the formation of degradation condensates [108].

#### LLPS regulation of the cGAS-STING pathway: innate immune activation and metabolic suppression

The cGAS-STING pathway is a core mechanism for cells to sense cytosolic DNA and initiate innate immune responses, playing a dual role in CRC: when normally activated, it promotes the production of type I interferons (IFNs) and enhances anti-tumor immunity; however, metabolic products in the TME often suppress this pathway [109–111]. LLPS is key to cGAS activation: when cytosolic DNA (such as from genomic instability or mitochondrial leakage) is present, cGAS forms liquid-like condensates through multivalent interactions with DNA via its positively charged N-terminal IDR, enhancing cGAS's enzymatic activity and 2′3'-cGAMP synthesis [112–114]. This further activates the STING-TBK1-IRF3 axis, recruiting immune cells (such as NK cells and CD8 + T cells). However, in the CRC tumor microenvironment, metabolic reprogramming leads to the accumulation of metabolites such as lactate and ketone bodies, which inhibit LLPS by modifying cGAS's PTMs. An innovative study shows that lactate induces lactylation of cGAS (such as at K384), disrupting its electrostatic complementarity with DNA, causing condensates to transition from a liquid to a gel state, reducing cGAMP production and IFN signaling. This weakens the antigen presentation function of myeloid cells (such as dendritic cells and macrophages), promoting immune evasion [115, 116]. Additionally, common microsatellite instability (MSI) in CRC leads to increased DNA damage, but cGAS condensates are suppressed and unable to effectively trigger immune responses [117]. Recent studies are exploring activation strategies, such as use of STING agonists (like ADU-S100) or lactate dehydrogenase inhibitors, to restore LLPS-mediated immune surveillance. STING agonists mimic cGAMP to activate the STING-TBK1-IRF3 axis, boosting type I interferon production and enhancing anti-tumor responses of CD8 + T cells and NK cells. However, metabolic suppression in the TME (e.g., lactate accumulation) can attenuate efficacy, necessitating combination with metabolic modulators (e.g., LDH inhibitors) to overcome resistance.

#### LLPS regulation of the p53 pathway: imbalance of tumor suppression and immune surveillance

P53 is a well-known tumor suppressor factor, mutated in approximately 50% of CRC cases, losing its transcriptional regulatory function [118]. LLPS is a core mechanism for p53 to exert its function: wild-type p53 undergoes phase separation through its C-terminal domain (including the DNA-binding domain and oligomerization domain), forming nuclear condensates that promote the transcription of downstream genes (such as p21 and PUMA), inducing cell cycle arrest and apoptosis [119]. In the CRC tumor microenvironment, this process is influenced by various factors. First, p53 mutations (such as R175H or R273H) enhance its LLPS tendency but lead to the transition of condensates from a liquid to a solid state, forming non-functional aggregates that not only lose tumor suppressive ability but also gain oncogenic functions, such as promoting EMT and chemoresistance. Furthermore, p53 dysregulation affects immune surveillance: under normal conditions, p53 condensates promote antigen presentation and T cell activation, but after mutation, it upregulates immune checkpoint molecules (such as PD-L1), recruiting Tregs [120–122]. The latest therapeutic strategies focus on restoring p53 function, such as using lactylation inhibitors (like β-alanine) or small molecule stabilizers (like APR-246), enhancing immunogenic cell death by modulating LLPS behavior [123–125]. APR-246 covalently modifies p53's DNA-binding domain (e.g., Cys124), restoring its phase separation capacity and activating transcription of apoptotic genes like PUMA. However, p53 mutant subtypes or lactylation-rich microenvironments may attenuate efficacy, necessitating combination with metabolic inhibitors (e.g., LDHA antagonists).

#### LLPS regulation of the TGF-β pathway: dual roles and cell context dependence

TGF-β signaling has dual roles in CRC: it acts as an early tumor suppressor in CRC by inhibiting cell proliferation and later promotes metastases and immunosuppression [126–128]. LLPS regulates the assembly of SMAD signal transduction complexes: Following TGF-β ligand binding and receptor activation, phosphorylated Smad2/3 proteins undergo LLPS with Smad4, driven by their IDRs. This leads to the formation of nuclear condensates that facilitate the binding to promoters of target genes such as *TAGLN* and *SNAI*, thereby regulating processes including apoptosis and differentiation [129, 130]. In the CRC tumor milieu, this process is further influenced by cell-specific TGF-β signaling responses. In CAFs, TGF-β signaling and the condensation of Smads augment fibrosis factor (COL1A1) gene expression. Thus, extracellular mechanical and extracellular matrices are re-modeled and indirectly impaired T cell infiltration in CRC [131]. In T cells, TGF-β signaling down-regulates cytotoxin functions of T cells and LLPS signaling and differentiates T cells into Tregs. In CRC, Smad4 mutations obviously impinge on condensation and lead to hyperactivation of TGF-β signaling and its increased promotion of tumors. In addition, LLPS in TGF-β signaling in CRC is further influenced by extracellular stress (hypoxia), which regulates TGF-β signaling in CRC. In this case, the process is mediated by HIF1α to promote angiomas and metastases. Other treatments are in use in TGF-β signaling signaling pathways and include inhibitors of TGFβ receptors and for small molecules that regulate LLPS condensation in Smads to counteract T cell immunosuppression [132–134].

### LLPS as a hub for matrix-immune crosstalk

#### Hub function initiation: metabolites disrupt innate immune surveillance by inhibiting cGAS phase separation

LLPS serves as an intracellular mechanism for the organization of molecules and can act as an intermediary for the complex integration of signals between stromal and immune cells, thus establishing a central point that connects the activities of different cell types in the TME [135]. In the TME of CRC, the interaction of stromal and immune cells occurs via several signaling pathways, thus inducing a malignant feedback loop for tumorigenesis [136–138]. LLPS can increase the efficiency of intercellular signaling by inducing the spatial coupling of signaling molecules.

Present-day research has implicated the cGAS–MITA/STING pathway as an essential nexus in YE and AT in which functionality depends on the phase-separated protein–protein complex that assembles when cGAS binds DNA. cGAS–DNA LLPS triggers the expression of downstream antiviral and antitumor effector genes [139–141]. By contrast, the carcinogenic metabolite acetaldehyde, which is abundant in CRC, inhibits cGAS–DNA binding and LLPS and severely reduces the functionality of the cGAS–MITA signaling cascade, suppressing AT responses and CD8 + T cell infiltration into tumors, leading to rapid tumor growth [142]. By implication, this observation highlights the fundamental importance of LLPS in and between stromal and YE cells and indicates that disruptions in LLPS-controlled signaling platform functionality can contribute to an oncogenic cycle of tumors by interfering with signaling integration in YE.

In addition, the enzyme for acetaldehyde metabolism, ALDH2, negatively correlates with the signals of immune activation in human colorectal cancer, and that higher expression of ALDH2 correlates with favorable prognosis for patients [143]. This apparent contradiction may arise because ALDH2's high expression could reflect a metabolic state that limits tumor aggressiveness or enhances DNA repair, thereby improving survival despite its stemness association, similar to how metabolic reprogramming in cancer influences prognosis through complex immune-microenvironment interactions [43]. Once more, it can be confirmed that LLPS-mediated integration of signals has an important consequence in reshaping the immune microenvironment of tumors.

#### Hub signal amplification: CAF phase separation shapes an immunosuppressive microenvironment through mechanical signal transmission

CAFs are known to contribute towards the generation of the immunosuppressive niche via LLPS-mediated mechanical signaling and are identified as a predominant component of the TME. It has been shown that phase separation in the CCS state leads to increased stiffness of the ECM, activation of YAP/TAZ signaling, and increased immunosuppressive factor secretion, making it easier for the tumors to evade immunity [144–146].

Mechanistically, CAFs produce and secrete large amounts of collagen, fibronectin, and other ECM proteins in the tumor tissues. They cause increased stiffness of the ECM due to crosslinking enzymes like LOX, causing dysfunctional and excessive mechano-signaling in the tumor tissues [147–149]. Stiffer ECM mechanoreceives and activates the YAP/TAZ signaling pathway in cancer cells and macrophages, in which YAP/TAZ act as vital co-transcription factors in regulating cell proliferation, migration, and immune regulatory gene expressions [150, 151]. In cancer cells, activated YAP/TAZ increases expressions of the immune-checkpoint molecules VISTA, suppressing antitumor functions of CD8 + T cells and enhancing immunosuppression [152]. Also, CAFs contribute to the promotion of M2 type immunosuppressive infiltration of tumor-associated macrophages in the tumor tissues due to cytokine and exosome releases, inducing the development of the YAP/TAZ signaling axis PP2A/STRN4, which represses STING/type I interferon signaling and reduces antitumor immunity [153]. Additionally, mechanical signaling transmission is increased by intracellular mechanical tension mediated by RhoA/ROCK/myosin-induced cytoskeletal contraction, leading to nuclear translocation and activation of YAP/TAZ, in turn establishing a self-perpetuating loop that further increases ECM production and stiffness, contributing to tumor progression [150]. In addition, CAFs' metabolite reprogramming, such as the induction of the glucose transporter GLUT1, maintain CAFs' activated and highly synthetic-metabolic state. From a therapeutic perspective, drug delivery systems mediated by exosomes have shown normalization of the CAFs' state, decreased stiffness, and increased infiltration of leukocytes, showing potential for use in the clinic [154, 155].

#### Hub bidirectional regulation: lactate drives p53 phase separation while promoting tumor progression and T cell exhaustion

Lactate, which is a primary output of cancer cell metabolic reprogramming, transduces p53 lactylation, leading to the induction of abnormal LLPS of p53 and modulation of tumor progression and T cell function suppression in both directions [156]. How this happens can be described as follows:

Firstly, the process of lactate-induced p53 lactylation is catalyzed by the aminoacyl tRNA synthetase enzyme AARS1, which serves as a lactate sensor and lactyltransferase. Lactate binds to and is converted to the lactate-AMP intermediate along with AMP, and this intermediate is then transferred to the lysine residues of p53, particularly residues 120 and 139 in the p53 DNA binding domain, affecting p53 structure and function and inhibiting its LLPS properties of binding and activating transcription, thus repressing p53’s tumor suppressor functions [157, 158]. Second, p53 lactylation and the resulting disruption of LLPS contribute to the promotion of tumorigenesis. In patients with cancers and wild-type p53, it has been observed that higher expression of AARS1 is positively correlated to higher levels of p53 lactylation and poor prognosis [159]. This indicates that lactate facilitates the survival and proliferation of cancer cells due to its role in regulating the phase separation and functions of p53.Thirdly, the role of lactate is not only within cancer cells but also in the immune microenvironment, as there are interactions between lactate and myeloid cells such as TAMs.

In the PTEN/p53 knockout model of cancers, lactate from cancer cells reduces phagocytic function of macrophages due to control of H3K18 lactylation in myeloid cells and supports an immunosuppressive microenvironment. Immune suppression mediated by lactate hinders antitumor immunity and supports the growth of tumors [160, 161].

In addition, lactate-mediated p53 lactylation and LLPS modulation also indirectly inhibits T cell function and proliferation. Lactate buildup in the tumor microenvironment leads to T cell metabolic dysfunction, thereby inhibiting T cell proliferation and cytolytic function, creating an immunosuppressive niche. In this manner, it further supports evasion of immunity and reduces the effectiveness of immunotherapy.

Lactate signaling and lactylation-related therapy has shown potential, for example, β-alanine inhibits p53 lactylation by suppressing the action of AARS1, and together with other pathways such as PI3K/MEK/Wnt, it can overcome the problem of immune suppression caused by macrophages in patients [160, 161]. By integrating inputs from metabolite-driven pathways, LLPS functions as a central organizer of multicellular signaling networks that perpetuate malignant cycles.

#### Hub system integration: phase separation coordinates multicellular signal networks to maintain malignant cycles

Homeostasis in the TME of CRC requires orchestrated intercellular communication, and LLPS is the organizing principle around which signaling from different dimensions is integrated to build a network that maintains the malignant matrix–immune equilibrium [162–164].

LLPS supports cross-cellular synchronized regulation through the use of signaling molecules (e.g., lactate), which inhibits cGAS LLPS (reduced innate immunity), disrupts p53 function (increased survival of tumor cells), and impairs T cell signaling microclusters (functional exhaustion), thus building an immune evasion network [89, 165, 166]. Simultaneously, one metabolite can regulate the LLPS of multiple core proteins, which can work in synchronization on both cancer and immune cells to build an optimal immune evasion network [167, 168]. Mechanical signals mediated by LLPS create positive feedback loops: for CAFs, LLPS leads to promotion of ECM stiffness, activation of YAP/TAZ LLPS and transcription, and induction of factors for fibrosis and immunosuppression (IL-6 and TGF-β), in turn fostering CAFs and ECM remodeling and supporting the malignant cycle [169]. LLPS further regulates the assembly of the transcription-initiating machinery and supports synchronized regulation of immunity: in M2-like cells of the TAM subset, YY1 condensates formed through phase separation simultaneously co-trigger the expression of factors for cancers (IL-6), rapidly promoting immunosuppression [170–172].

LLPS critically orchestrates immune cell infiltration in colorectal cancer by coordinating dysfunction across stromal and immune cells. Directly, LLPS disruption in T cells (e.g., impaired LAT/GRB2 signaling clusters) causes functional exhaustion. Indirectly, LLPS in stromal cells creates barriers: endothelial LLPS drives abnormal angiogenesis, while metabolite-altered LLPS in myeloid cells (e.g., lactate-suppressed cGAS activity) blunts innate immunity. Thus, LLPS integrates these mechanisms to sustain an immune-excluded microenvironment (Table 2).

**Table 2:** Key LLPS events in the CRC TME and their functions in the stromal-immune

Key Cell Type	Core LLPS Components/Process	Upstream Regulators/Inputs	Impact on Stromal-Immune Axis	Pro-Tumorigenic Outcome	Key References
Cancer-Associated Fibroblasts (CAFs)​	Transcriptional coactivator condensates (e.g., YAP/TAZ)	Increased ECM stiffness, TGF-β signaling	Promotes secretion of immunosuppressive factors (e.g., IL-6, TGF-β) and ECM remodeling, recruiting Tregs/MDSCs and inhibiting CD8 + T cell infiltration	Sustained CAF activation, immunosuppression, and tumor progression	[35, 83–86]
Tumor-Associated Macrophages (TAMs)​	Lactate-induced HIF-1α nuclear condensates	High lactate levels from tumor glycolysis	Enhances HIF-1α transcriptional activity, driving M2 polarization and expression of immunosuppressive genes (e.g., IL-10, ARG1)	Innate immune evasion, establishment of an immunosuppressive niche	[53–55]
Myeloid-Derived Suppressor Cells (MDSCs)​	Transcriptional condensates for ARG1/iNOS expression	Inflammatory signals in the TME	Upregulates expression of key immunosuppressive enzymes, directly inhibiting T cell function	Suppression of adaptive anti-tumor immunity	[59, 60]
CD8 + T Cells​	T cell receptor (TCR) signaling clusters (e.g., LAT, GRB2)	TCR activation by antigen	Normal:​ Facilitates efficient T cell activation. CRC TME:​ Disrupted by metabolites (lactate, PGE2) and PD-L1/PD-1 interaction	Functional impairment or exhaustion of cytotoxic T cells	[65, 68, 72]
Tumor Cells/Immune Cells​	cGAS-DNA condensates	Cytosolic DNA (e.g., from genomic instability)	Normal:​ Activates cGAS-STING pathway and anti-tumor immunity. CRC TME:​ Inhibited by lactate-induced lactylation of cGAS	Immune evasion via suppression of innate immune sensing	[75, 100, 103]
Tumor Cells (p53 wild-type)​	p53 transcriptional condensates	Cellular stress signals (e.g., DNA damage)	Normal:​ Transactivates tumor suppressor genes and contributes to immune surveillance. CRC TME:​ Disrupted by lactate-induced lactylation, impairing p53 function	Loss of tumor suppression and compromised immune surveillance	[108, 109]

#### The translational challenge and paradigm of targeting LLPS

The delineation of LLPS as a master regulator of the malignant stroma-immune axis solidifies its therapeutic appeal. However, translating this appeal into viable strategies is confounded by a fundamental biological duality: LLPS is a ubiquitous physicochemical mechanism essential for both physiological homeostasis and pathological reprogramming [173]. This intrinsic duality creates a critical targeting dilemma. For example, while LLPS is required for the tumor-suppressive activity of wild-type p53 and the pro-inflammatory signaling of cGAS, the same process is co-opted to stabilize oncogenic β-catenin/TCF complexes and form dysfunctional mutant p53 aggregates in CRC [174, 175]. Therefore, the therapeutic objective must evolve from broadly inhibiting phase separation to the precise disruption of context-defined pathogenic condensates.

This necessitates a paradigm of “precision condensate intervention,”where therapeutic agents are designed to discriminate pathological assemblies from their physiological counterparts. Selectivity could be achieved by targeting unique, disease-specific molecular features that define the pathogenic condensate. This approach is exemplified by the cataloging of cancer-type-specific LLPS drivers across malignancies, such as EML4-ALK condensates in lung adenocarcinoma [176], mutant SPOP compartments in prostate cancer [177], and FUS-CHOP oncogenic condensates in myxoid liposarcoma [178]. These examples represent distinct compositional signatures—such as mutant protein interfaces, tumor-specific post-translational modifications, or aberrant physicochemical properties—that constitute potential therapeutic vulnerabilities for selective disruption. Identifying such distinct features is paramount. The following sections evaluate emerging strategies—small molecule inhibitors, protein degraders, and nanodelivery systems—through this lens of requisite selectivity, assessing their potential to overcome this central translational challenge.

### Targeting pathogenic LLPS condensates: a new frontier in reversing matrix-immune axis imbalance

**Fig. 5:**
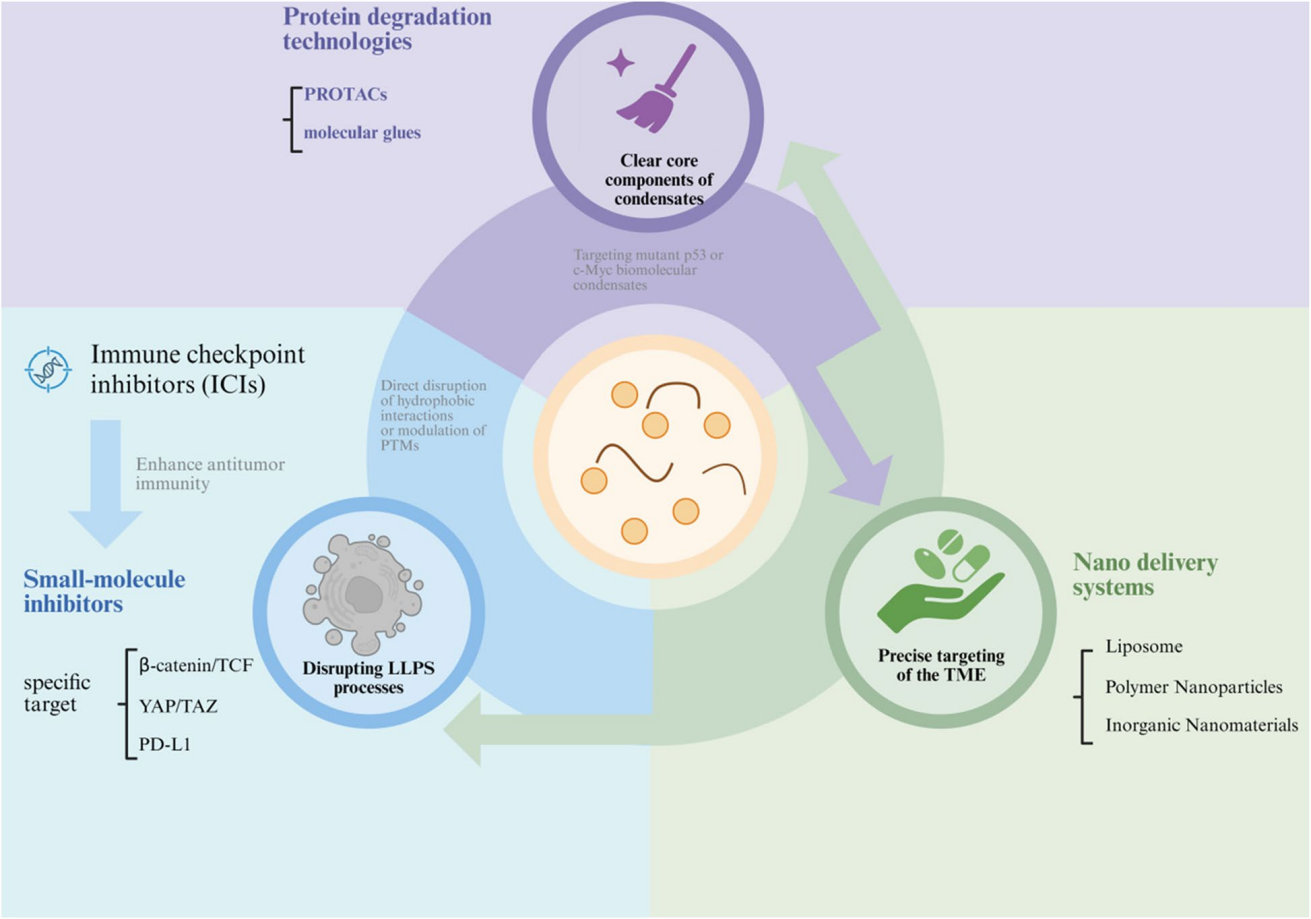
Synergistic therapeutic strategies targeting liquid–liquid phase separation. This illustration systematically outlines three categories of synergistic therapeutic strategies targeting LLPS, all aiming at disrupting pathogenic biomolecular condensates. Protein degradation technologies​ (left) utilize PROTACs and molecular glues to specifically recognize and clear core components of aberrant condensates (e.g., mutant p53 or c-Myc), dismantling the pathogenic condensates at their source. Direct intervention strategies​ (top) combine small molecule inhibitors (targeting LLPS-associated pathways like β-catenin/TCF and YAP/TAZ) with immune checkpoint inhibitors (e.g., anti-PD-L1). These act by directly disrupting hydrophobic interactions or modulating post-translational modifications to dissolve pathogenic condensates, while simultaneously enhancing anti-tumor immunity. Nano delivery systems​ (right) employ carriers such as liposomes and polymer nanoparticles to achieve precise drug delivery within the tumor microenvironment, improving efficacy and reducing toxicity. These three strategies, working in synergy (indicated by arrows), form a multi-dimensional therapeutic system targeting LLPS, offering a novel paradigm for overcoming tumor drug resistance

As discussed, LLPS has been identified as an important target as fuels the malignant conversation of the matrix–immune axis in the TME of CRC. Based on this understanding, focusing on LLPS condensates as pathological lesion constitutes a novel approach that aims to override the immunosuppressive state and interrupt the process of tumorigenesis. In this section, three innovative approaches focused on this axis will be described in detail: small molecule inhibitors, protein degradation tools, and nanosystems for delivery, signifying a multi-layered approach that stretches from “formation inhibition” toward “complete removal” and finally toward “targeted delivery" (Fig. 5).

#### Small molecule inhibitors: interfering pathogenic phase separation processes

LLPS, as a key physicochemical process regulating the formation of intracellular biomolecular condensates, is closely related to the occurrence and development of various diseases, including CRC. In recent years, small molecule inhibitors targeting pathogenic condensates have become a research hotspot in targeted therapy, providing innovative strategies for intervening in the malignant dialogue of the matrix-immune axis in the CRC tumor micro-environment by specifically interrupting the LLPS process [179]. Small molecule inhibitors mainly interfere with LLPS by directly disrupting the hydrophobic interactions that mediate phase separation or regulating PTMs of proteins [180]. For example, G3Ia/G3Ib inhibits stress granule formation by binding to G3BP1/2 [181] phosphorylation modifications of Axin regulate its phase separation behavior, thereby affecting the Wnt pathway [164] and curcumin and other multi-target inhibitors demonstrate broad-spectrum potential based on hydrophobic interactions [182, 183]. These mechanisms provide a theoretical basis for targeting abnormal condensates.

In CRC, small molecule inhibitors can precisely target key pathogenic condensates: targeting β-catenin/TCF condensates (e.g., by regulating Axin or directly interfering β-catenin oligomerization) can inhibit Wnt pathway-driven tumor proliferation [164]; inhibiting the formation of YAP/TAZ transcriptional activation condensates can block their tumor-promoting functions [166]; intervening in the assembly of PD-L1 transcriptional regulatory condensates can reverse T cell exhaustion [162]. Notably, some small molecules (such as curcumin) possess polypharmacological properties, simultaneously affecting multiple targets and providing synergistic treatment opportunities for CRC [165].

The combination of small molecule inhibitors with existing therapies shows synergistic potential:combining with Wnt pathway inhibitors can enhance the inhibitory effect on β-catenin/TCF condensates [181]; combining with immune-checkpoint inhibitors (ICIs) can improve the immune micro-environment and enhance efficacy by mechanisms such as reducing PD-L1 expression [179, 184]. The excellent cell membrane permeability of small molecule inhibitors helps overcome TME delivery bottlenecks, and their structural modifiability also provides space to overcome resistance. Future research should focus on dose optimization, administration timing, and biomarker-based patient stratification to promote clinical translation.

While the targeting of biomolecular condensates by small molecules holds considerable therapeutic promise, their clinical translation faces substantial challenges. Most inhibitors—such as verteporfin, which targets YAP—demonstrate efficacy in preclinical models but may fail in human trials due to off-target effects, such as unintended disruption of LLPS in normal cells. For instance, setbacks encountered with TGF‑β inhibitors like galunisertib underscore the necessity of thoroughly evaluating the toxicity profiles of LLPS-targeting agents. Key unresolved questions persist, particularly concerning the rational design of context-selective modulators, as well as the imperative to integrate biomarkers—such as imaging-based detection of LLPS condensates—for patient stratification and response prediction.

#### Protein degradation technologies: clearing core components of condensates

**Fig. 6:**
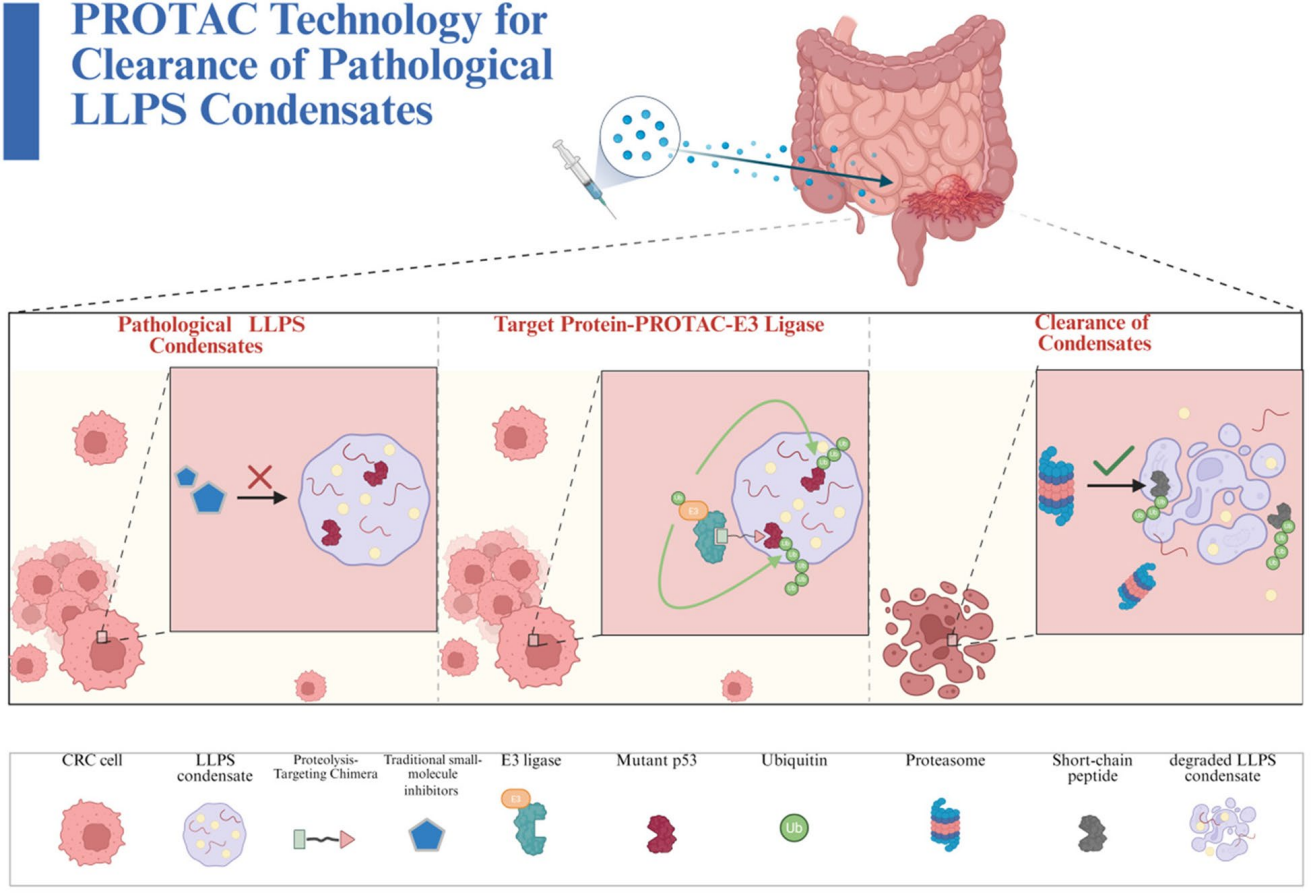
Mechanism of PROTAC-mediated clearance of pathological LLPS condensates. This figure schematically illustrates the targeted degradation of LLPS condensates via PROTAC (Proteolysis-Targeting Chimera) technology, a novel therapeutic strategy for CRC. Panel 1 (Pathological LLPS Condensates):​ The left panel depicts the problem: a CRC cell harbors a pathological LLPS condensate (e.g., formed by mutant p53). Traditional small-molecule inhibitors (blue pentagon) fail to clear this condensate, as indicated by the red "X", highlighting the limitation of conventional therapeutics. Panel 2 (Target Protein-PROTAC-E3 Ligase Complex):​ The central panel elucidates the core mechanism. A heterobifunctional PROTAC molecule (blue-green) acts as a molecular bridge. It simultaneously engages a target protein within the condensate (e.g., mutant p53, red) and an E3 ubiquitin ligase (teal). This recruitment brings the E3 ligase into proximity with the target protein, facilitating its ubiquitination (green circles).Panel 3 (Degradation and Clearance):​ The right panel demonstrates the successful outcome. The ubiquitinated target protein is recognized and degraded by the proteasome, yielding short-chain peptides. The disintegration of the pathological LLPS condensate into a degraded state, confirmed by the checkmark, signifies the restoration of normal cellular protein homeostasis. Summary:​ This triptych visually summarizes the key advantage of PROTACs—achieving event-driven, potent degradation of pathogenic biomolecular condensates that are recalcitrant to traditional occupancy-based inhibitors, offering a promising strategy for targeted protein degradation in CRC

Protein degradation technologies (such as PROTACs and molecular glues) provide innovative strategies for targeting pathogenic LLPS condensates (such as mutant p53) in CRC by inducing pathogenic proteins to enter the ubiquitin–proteasome system for degradation, dismantling condensates and blocking matrix-immune axis functions, showing therapeutic potential [185–187] (Fig. 6) (Table 3).

**Table 3:** Comparative analysis of therapeutic strategies targeting pathogenic LLPS

Therapeutic Modality	Representative Approaches/Mechanisms	Key Advantages	Major Challenges/Limitations	Clinical Translation Perspective & Combinatorial Potential	Key References
Small Molecule Inhibitors​	- Direct condensation disruptors: Interfere with multivalent interactions (e.g., targeting YAP/TAZ or β-catenin/TCF condensates) - PTM modulators: Regulate post-translational modifications that influence LLPS	- Favorable pharmacokinetics (e.g., good cell membrane permeability) - Potential for oral administration - Amenable to rational chemical optimization	- Potential lack of specificity due to the dynamic nature of condensates - Risk of acquired resistance - Requires deep understanding of target condensate's physical chemistry	- Combination with ICIs: To reverse specific immunosuppressive pathways (e.g., by reducing PD-L1 expression) - Patient stratification​ based on specific LLPS-related biomarkers is crucial	[162, 164–166]
Protein Degradation Technologies (PROTACs, Molecular Glues)​	- PROTACs: Bifunctional molecules recruiting target protein to E3 ubiquitin ligase for ubiquitin–proteasome degradation - Molecular Glues: Induce novel protein–protein interactions leading to target degradation	- Can target "undruggable" proteins that lack enzymatic activity - Catalytic mode of action offers sustained effect - Potential to completely ablate oncogenic condensates by removing core components	- Large molecular size may limit tissue penetration and oral bioavailability - Requires efficient engagement of specific E3 ligases within the TME - Potential for on-target off-tumor toxicity	- Potentiating immunotherapy: e.g., Degrading BET proteins to induce immunogenic cell death - Dual-targeting PROTACs​ (e.g., targeting β-catenin and STAT3) to overcome redundancy	[96, 167–171, 177]
Nano-Drug Delivery Systems​	- Smart carriers: e.g., pH-responsive, enzyme-responsive, or redox-responsive nanoparticles - Targeted delivery: Surface-functionalized with ligands for active targeting of TME or specific cells	- Enhances drug solubility and bioavailability - Reduces systemic toxicity via targeted accumulation (EPR effect). < br.- Can be engineered for co-delivery of multiple therapeutic agents (e.g., drug + inhibitor)	- Complexity in manufacturing and scalability - Potential immunogenicity and long-term biocompatibility concerns - Tumor heterogeneity may affect delivery efficiency	- Ideal platform for combination therapies: Co-delivering LLPS-targeting agents with chemo/immunotherapy drugs - Precision targeting: Using TME-specific stimuli to trigger drug release, maximizing efficacy and minimizing side effects	[187, 190, 192, 196, 202]

PROTACs promote ubiquitination and degradation by connecting target proteins with E3 ligases through multivalent molecules. For example, NP-PROTACs can dual-degrade β-catenin and STAT3, enhancing anti-tumor effects [108]. PROTACs mediate ubiquitination and degradation of pathogenic LLPS proteins (e.g., mutant p53) via E3 ligases (e.g., VHL), dismantling condensate cores. However, their large molecular size impedes tumor penetration, and E3 ligase heterogeneity may drive resistance. Dual-targeting PROTACs or nanodelivery systems could enhance specificity and synergize with immunotherapy. PROTAC-mediated degradation of BET proteins can induce immunogenic cell death and improve immune therapy responses [188, 189].

Molecular glue technology achieves specific ubiquitination and degradation of target proteins by promoting unnatural interactions between target proteins and E3 ubiquitin ligases [187, 190, 191]. This technology is also applicable for targeting pathogenic phase separation proteins, providing an effective supplement, especially in cases where PROTACs are difficult to target [192–194]. Through such technologies, it is possible to destroy nucleic acid-binding proteins or transcriptional regulators that form phase-separated condensates, thereby blocking cancer signaling pathways and inhibiting tumor progression.

The application of protein degradation technologies in CRC faces challenges: the dynamics and spatial heterogeneity of pathogenic condensates hinder the penetration of degradants; differences in the expression and activity of E3 ligases increase the difficulty of designing specific drugs [195]; tumor heterogeneity and compensatory resistance mechanisms may weaken efficacy [196–198]. In addition, LLPS condensates regulate protein accessibility [199], thus reaffirming the role of degradants and depolymerization adjuvants that can penetrate LLPS condensates. Based on methodologies like nano-delivery systems, multi-pathway interventions, and mechanism explorations, efforts are put towards overcoming barriers pertaining to penetrability, specificity, and resistance.

In this manner, to overcome such hurdles,optimalization of the molecular structure of the protein degradant for improving cell permeability and specific delivery to the tumor tissue can be identified as an important aim. Thus, the formulation of nano-delivery systems (NP-PROTACs) can improve the stability and specific delivery of degradants within the tumor microenvironment, leading to better effectiveness of the degradant in degrading pathogenic proteins that cause phase-separation disorders. In addition, the dual-degradation approach targeting both the ubiquitin–proteasome system and the autophagy-lysosome system can be expected to counteract potential compensatory effects caused by single-degrading pathways, leading to improved degrading efficiency and antitumor effects [200]. In relation to target candidate selection, it can be hypothesized that the validation of specific proteins closely related to phase-separation and condensate-related functions (for example, the role of TRIM55 in regulating c-Myc [201] and the role of UBD in p53 degradation [202]) can contribute to the specific creation of degrading systems.

Some of the potential areas of future work include: the creation of degradants that use properties of LLPS (for instance, binding phase-separation conformations or triggering depolymerization) [203], use of nanotechnology (for example, PSETACs for delivering mRNA) [204]; the development of multi-target degradants in attempt to overcome resistance; and multi-omics analysis in combination with microscopy analysis for gaining insights into the mechanism and investigating potential synergy between degradants and immunotherapy [108].

#### Nano-delivery systems: precisely targeting the tumor micro-environment

Because nanomaterials have characteristic physicochemical properties, it holds great potential for specific targeting of phase-separated condensates in the TME. By well-designed carrier systems, it is possible to achieve specific delivery and controlled release of pathogenic condensates and, in turn, regulate the matrix-immune axis and pathological conditions [205–207].

In nanocarriers for intelligent delivery, the reliance on TME-based environmental factors (e.g., pH, enzymes, redox state) for targeted drug delivery is particularly important [208, 209]. Common types of nanocarriers include liposomes, polymeric nanocarriers, and inorganic nanocarriers, which can be steered towards cell receptors or condensates based on ligand conjugation methods.Optimization of nanocarrier size, shape, and charge enables functional customization: pH-sensitive versions release drugs in acidic microenvironments [210, 211]; enzyme-responsive designs exploit tumor-associated enzymes for enhanced uptake and retention [212, 213]; and engineered polymeric carriers integrate multiple stimuli responses for selective targeting of condensates and pathways.

Nanocarriers greatly promote drug delivery efficiency, improving drug stability and bioavailability in vivo and reducing non-specific distribution and systemic toxicities compared to conventional treatments [214, 215]. For example, PLGA-PEG nanospheres and nanocapsules exhibit little effect on platelet function, ensuring the biosafety of the drug delivery system itself [214]. Liposomes and polymeric nanospheres can obviously prolong circulation half-life and overcome immune clearance via PEGylation and ligand modification, further improving both passive and active tumor-targeting efficiency [216, 217]. Nanocarriers can strongly penetrate the stroma of tumors and overcome increased interstitial fluid pressure and high density of stromal cells for intratumoral distribution and drug delivery. In addition, drug delivery can be achieved in a controlled manner, such as pH or enzyme responsiveness, further improving systemic toxicities and drug safety [218, 219]. Advances in multi-target and multi-drug synergistic nanoplatforms aim to counteract the complicated and multi-factorial nature of tumors and resistance [220].

In this manner, nanotechnology has shown potential for pathogenic condensate targeting, especially in the areas of precise drug delivery and minimizing side effects [221], including the use of findings from neurodegenerative disorders [222]. However, translation into medical practice faces several obstacles for nanomaterials in terms of compatibility and validation for use in tumors and TME, which demand very precise on-targeting abilities [223, 224]. In the future, it is advised that imaging and responsive systems be combined for intelligent and cell-engineering-based approaches for tailored therapies [225, 226].

## Conclusion and challenges

This review critically and comprehensively describes the role of LLPS in the TME of CRC. It asserts that LLPS doesn't regulate any single cell function in isolation, but instead orchestrates the activation and tumorigenic functions of CAFs, dysfunctional angiogenesis of ECs, and exhaustion of myeloid cells and T cells in forming an extremely integrated dynamic network.

LLPS fundamentally reshapes the immune contexture of the CRC TME by integrating stromal-immune axis dysfunctions. It drives immunosuppression through coordinated mechanisms: in stromal cells, LLPS promotes fibrosis and aberrant angiogenesis that physically impede immune infiltration; in immune cells, it directly disrupts functional competence, such as by impairing T-cell receptor signaling and polarizing myeloid cells toward inhibitory phenotypes. This collective rewiring establishes a self-sustaining, immune-excluded niche that underpins tumor evasion and progression.Findings from this review generally point out that LLPS is an underlying platform linking malignant cross-talk between the extracellular-matrix and immunity in a paradigm that doesn't just use linear signaling in understanding the TME of CRC.

Notwithstanding, the translational path of LLPS-targeting therapies is fraught with challenges that must be acknowledged. First, the field's heavy reliance on reductionist in vitromodels (e.g., monolayer cell cultures) fails to recapitulate the spatial heterogeneity and multicellular crosstalk of the native tumor microenvironment (TME). This limitation obscures the true complexity of LLPS regulatory networks and hinders the clinical predictive value of preclinical data. Second, current pharmacological modalities face inherent hurdles: small-molecule inhibitors often lack specificity for dynamic condensates, while advanced technologies like PROTACs and nanocarriers grapple with delivery efficiency and potential on-target off-tumor toxicity. Perhaps most critically, the inherent adaptability of the TME—epitomized by LLPS-driven compensatory circuits such as the CAF-ECM-YAP stiffness feedback loop—poses a fundamental risk of treatment resistance, demanding a shift from monotherapy to rational combination strategies.

Overcoming these hurdles mandates a multifaceted future effort. Mechanistically, we must prioritize the integration of advanced in vivoimaging and multi-omics approaches (e.g., spatial transcriptomics, proteomics) to map the LLPS 'interactome' within intact tissues, enabling patient stratification and the identification of predictive biomarkers. Technologically, the focus should be on developing next-generation therapeutics with enhanced specificity and delivery. This includes engineering context-sensitive degraders (e.g., dual-targeting PROTACs) and 'smart' nanoplatforms that co-deliver LLPS disruptors (e.g., YAP/TAZ or β-catenin condensate inhibitors) with established immunotherapies (e.g., immune checkpoint blockers). Such combinatorial strategies, designed to simultaneously disrupt the stromal and immune axes of the TME, represent the most promising avenue to overcome compensatory resistance and achieve durable anti-tumor responses.Ultimately, a deeper understanding of LLPS as the pivotal coupler of the CRC matrix-immune axis not only refines our mechanistic knowledge of malignant progression but also illuminates a clear path for developing novel, multidimensional therapeutic strategies.

## Data Availability

No datasets were generated or analysed during the current study.
